# Supplementary material for: Discovery and characterization of gene-by-environment and epistatic genetic effects in a vertebrate model
Source: Cell Genom. 2026 Feb 10;6(5):101164. doi: 10.1016/j.xgen.2026.101164 (PMC13174225; doi:10.1016/j.xgen.2026.101164)
Supplement: Document S1. Figures S1–S10 and Tables S1–S3 [file mmc1.pdf]

Cell Genomics, Volume *6*

Supplemental information

Discovery and characterization of gene-by-environment and epistatic genetic effects in a vertebrate model

Bettina Welz, Saul Pierotti, Tomas Fitzgerald, Thomas Thumberger, Risa Suzuki, Philip Watson, Jana Fuss, Tiago Cordeiro da Trindade, Fanny Defranoux, Marcio Ferreira, Kiyoshi Naruse, Felix Loosli, Jakob Gierten, Joachim Wittbrodt, and Ewan Birney

Cross name	Female founder strain	N. of females founders	Male founder strain	N. of male founders	N. of F1 fish	N. of F2 embryos
IP (72-2 x 79-2)	IP 79-2	3	IP 72-2	1	36	199
IP (72-2 x 15-1)	IP 15-1	1	IP 72-2	1	35	194
IP (72-2 x 139-4)	IP 139-4	1	IP 72-2	1	4	181
IP (72-2 x 55-2)	IP 55-2	3	IP 72-2	1	21	591
IP (72-2 x 62-2)	IP 62-2	4	IP 72-2	1	34	208
IP (72-2 x 68-1)	IP 68-1	4	IP 72-2	1	40	162
IP (139-4 x 72-2)	IP 72-2	3	IP 139-4	1	37	167
IP (72-2 x 22-1)	IP 22-1	2	IP 72-2	1	15	169
IP (68-1 x 79-2)	IP 68-1	4	IP 79-2	1	34	181
IP (55-2 x 139-4)	IP 55-2	2	IP 139-4	1	29	426
IP (15-1 x 62-2)	IP 62-2	5	IP 15-1	1	12	189

Table S1. Overview of the MIKK panel strain crosses used for segregation analysis, related to Figure 2. Number (N.) of cross founder individuals, F1 mating groups sizes and number of F2 embryos heart rate phenotyped per cross. IP: Inbred Panel.

Gene symbol	ENSEMBL gene ID	gRNA target site [PAM]	Oligo sequence 5' to 3' direction (PCR forward primer)	Oligo sequence 5' to 3' direction (PCR reverse primer)
ryr2b	ENSORLG00000005221	TCAATCTTCAACTGAC AAGC[TGG]	TTGTGAGGTCTGACTGG CTTT	ACCTCCATCACCAAC CACCT
ccdc141	ENSORLG00000030409	CGAGAACGAGGTTTTA GCTG[TGG]	ACACAAGTCACAGGGAT CAGTT	GTACCTCTGTGGCTT GTCGG
ppp3cca	ENSORLG00000016121	AGCATTTCCGTCACTG TGCA[AGG]	TGTGTTTGTGGCTCATT TGCA	TCTAAGCTAACATCA GATCAGGTT
sptbn1	ENSORLG00000002006	CCATCCAGGCCGACC GCGTC[CGG]	AAACATGGTTGGAGCTG CATG	ACAATATCTTGAAGG GACAGCG
atg7	ENSORLG00000004519	GAGACACAAGCTGCA GAGGT[AGG]	GGTCTTGGACAACTATG AGAAAGA	GAATCACTGGTACCC TGCCC

Table S2. Oligo and gRNA sequences used to amplify and target the selected candidate genes, related to Figure 5. Locus-specific CRISPR/Cas9 and base editor target sites with the PAM of the gRNA sequence in brackets and oligonucleotides used for target site amplification via PCR.

Generative model	Discovery model	Env. noise (%)	r^2 to causal variant	Discoverable QTLs (of 14)
With GxG and GxGxE	Same as generating model	0	1	14
With GxG and GxGxE	G + E + GxE	0	1	13
With GxG and GxGxE	G + E + D	0	1	12
With GxG and GxGxE	G + E	0	1	12
With GxG and GxGxE	G + E	1	1	11
With GxG and GxGxE	G + E	10	1	5
With GxG and GxGxE	G + E	50	1	1
With GxG and GxGxE	G	0	1	0
With GxG and GxGxE	Same as generating model	0	0.9	0
With GxG and GxGxE	G + E + GxE	0	0.9	0
With GxG and GxGxE	G + E + D	0	0.9	0
With GxG and GxGxE	G + E	0	0.9	0
With GxG and GxGxE	G + E	1	0.9	0
With GxG and GxGxE	G + E	10	0.9	0
With GxG and GxGxE	G + E	50	0.9	0
With GxG and GxGxE	G	0	0.9	0
No GxG and GxGxE	Same as generating model	0	1	12
No GxG and GxGxE	G + E + GxE	0	1	12
No GxG and GxGxE	G + E + D	0	1	11
No GxG and GxGxE	G + E	0	1	11
No GxG and GxGxE	G + E	1	1	11
No GxG and GxGxE	G + E	10	1	2
No GxG and GxGxE	G + E	50	1	1
No GxG and GxGxE	G	0	1	0
No GxG and GxGxE	Same as generating model	0	0.9	0
No GxG and GxGxE	G + E + GxE	0	0.9	0
No GxG and GxGxE	G + E + D	0	0.9	0
No GxG and GxGxE	G + E	0	0.9	0
No GxG and GxGxE	G + E	1	0.9	0
No GxG and GxGxE	G + E	10	0.9	0
No GxG and GxGxE	G + E	50	0.9	0
No GxG and GxGxE	G	0	0.9	0

Table S3. Simulation summary, related to Figure 6. Summary of the simulation results described in **Figure 6**. A QTL is deemed discoverable if the minimum sample size required to discover it at p < 5*10-8 is smaller than 500K for an allele frequency smaller than 1%.

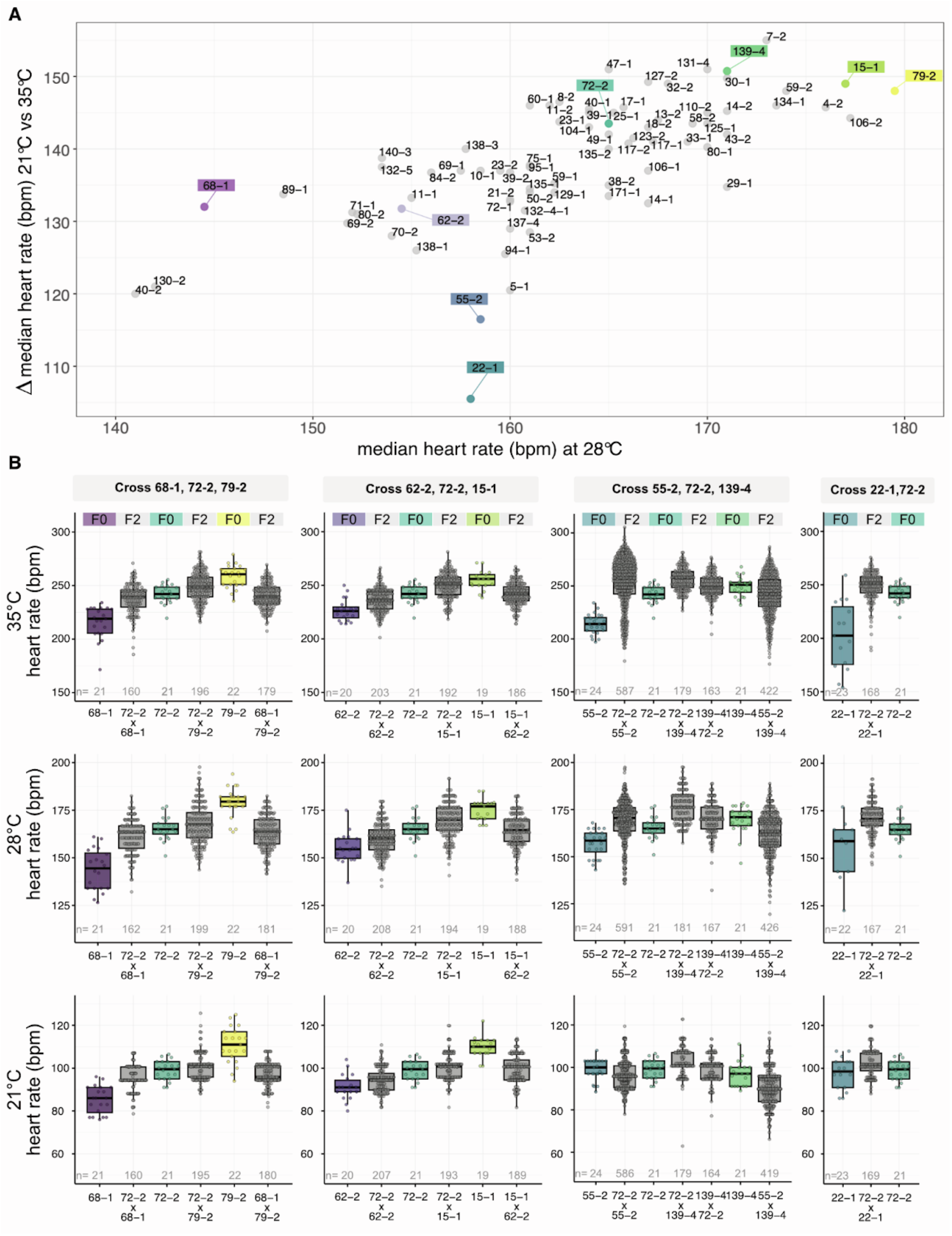

Figure S1. Strain-dependent heart rate change in the MIKK panel in response to temperature and subsequent heart rate segregation by 11 different crosses, related to Figure 2. A: Heart rate change from 21°C to 35°C and baseline heart rate at 28 °C varies across the MIKK panel strains, indicating different temperature-response characteristics. Strains selected for segregation analysis are highlighted. **B:** Heart rate of recombinant F2 embryos (grey) in comparison to the heart rate of the parental strains (F0) across the measured temperatures. Data is displayed as box plots (median +/- interquartile range between the 25th and 75th percentiles) and overlaid scatter plots of individual heart rate measurements (n) in beats per minute (bpm).

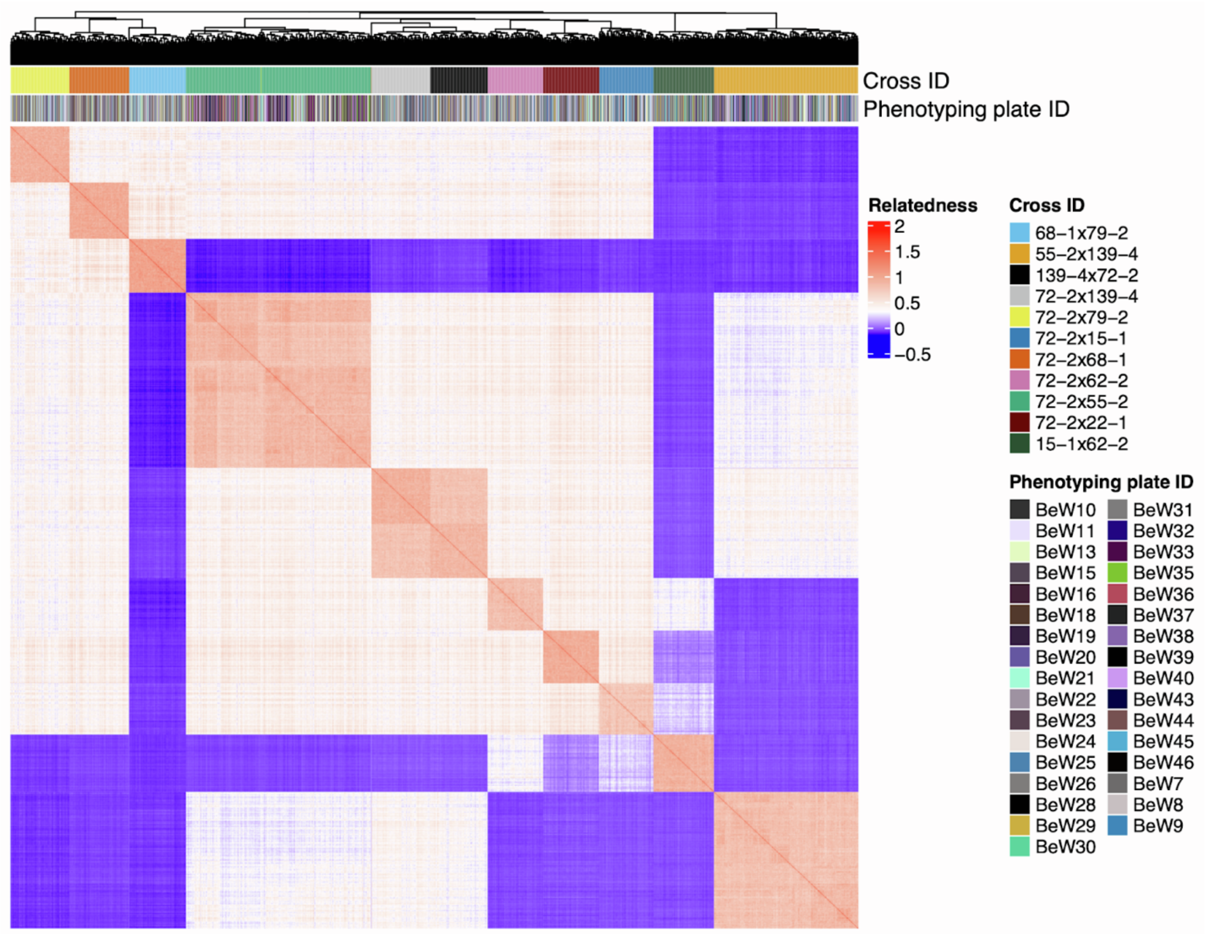

Figure S2. Genetic relatedness matrix of F2 samples, related to Figure 3. Heatmap of pairwise genetic relatedness among the samples of the multi-parental F2 population used in the GWAS analysis described in this work. Rows and columns are clustered by genetic relatedness, demonstrating spontaneous clustering of the samples belonging to the same F2 cross ("cross ID" annotation). The "Phenotyping plate ID" annotation demonstrates the randomisation achieved between phenotyping batches and population structure. Note the sub-clustering within the reciprocal cross (72-2 x 139-4 and 139-4 x 72-2), highlighting shared parental genetic contributions.

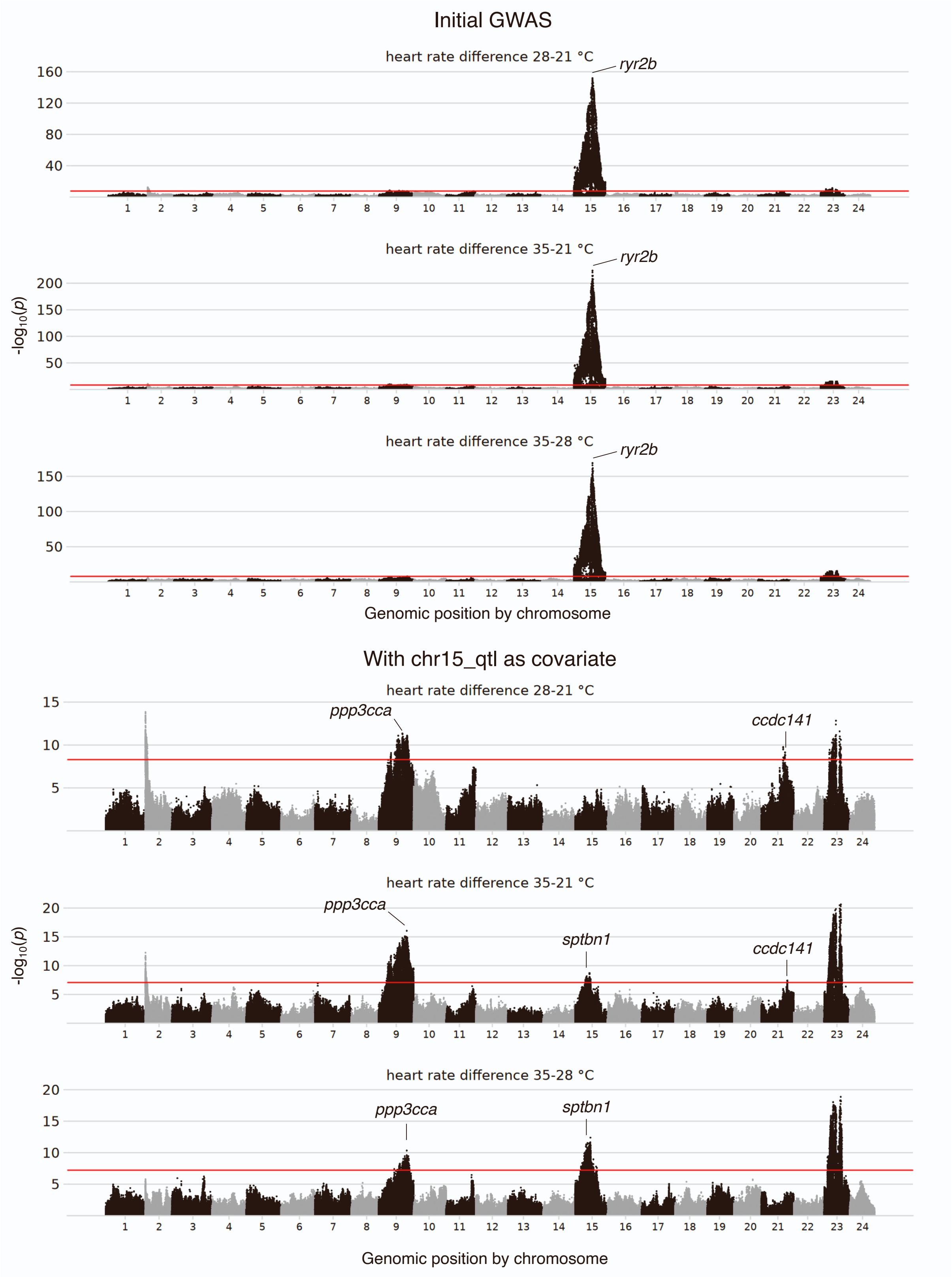

Figure S3. Manhattan plots of the heart rate temperature response phenotypes, related to Figure 3. Manhattan plots for the association of the difference in heart rate across temperature treatments with genetics. The significance threshold (red line) corresponds to the minimum *p*-value achieved over 100 permutations. The genes selected for experimental validation are indicated. In the bottom three panels, the strong locus at chromosome 15 was included as a covariate to unmask weaker effects. *p*-values: likelihood-ratio test.

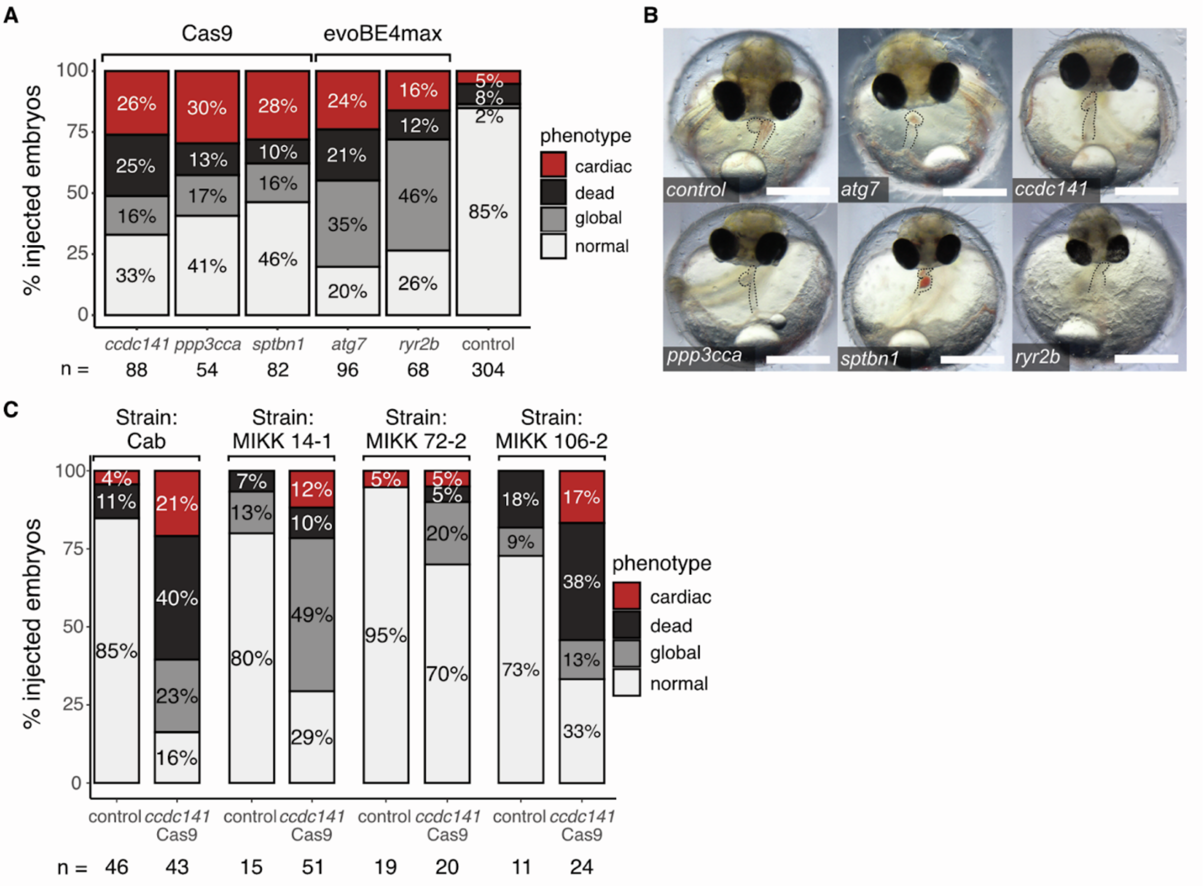

Figure S4. Phenotype proportions after gene editing of candidate genes, related to Figure 5. A: Phenotype proportions after CRISPR/Cas9- and base editor - mediated gene editing of five selected candidate genes reveals increased numbers of cardiac affected embryos 4 days post injection compared to the mock-injected control. **B:** Representative cardiac affected crispants and editants in comparison to normal developed control embryo (scale bar: 500µm). **C:** Phenotype proportions after CRISPR/Cas9-mediated genome targeting of *cccdc141* in four different medaka strains in comparison to mock-injected controls. n: number of injected embryos.

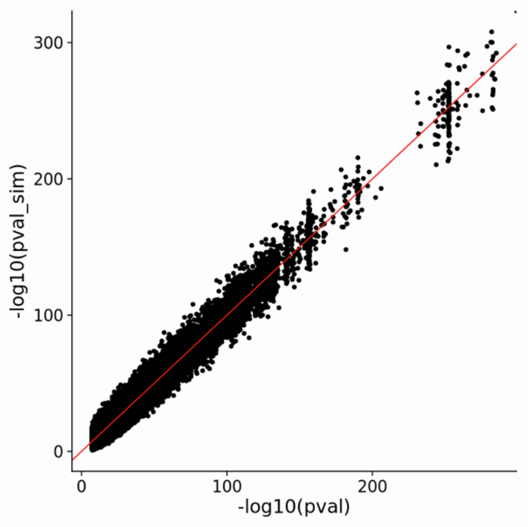

Figure S5. Simulation example in the UK Biobank, related to Figure 6. Correlation between p-values obtained from the Neale lab UK Biobank GWAS for human height (https://www.nealelab.is/uk-biobank) and p-values for the same markers generated using an adapted version of our simulation approach, parameterised with effect sizes and residual variances from the same dataset (see STAR Methods). The two sets of p-values are highly correlated and show no systematic bias. *p*-values: likelihood-ratio test.

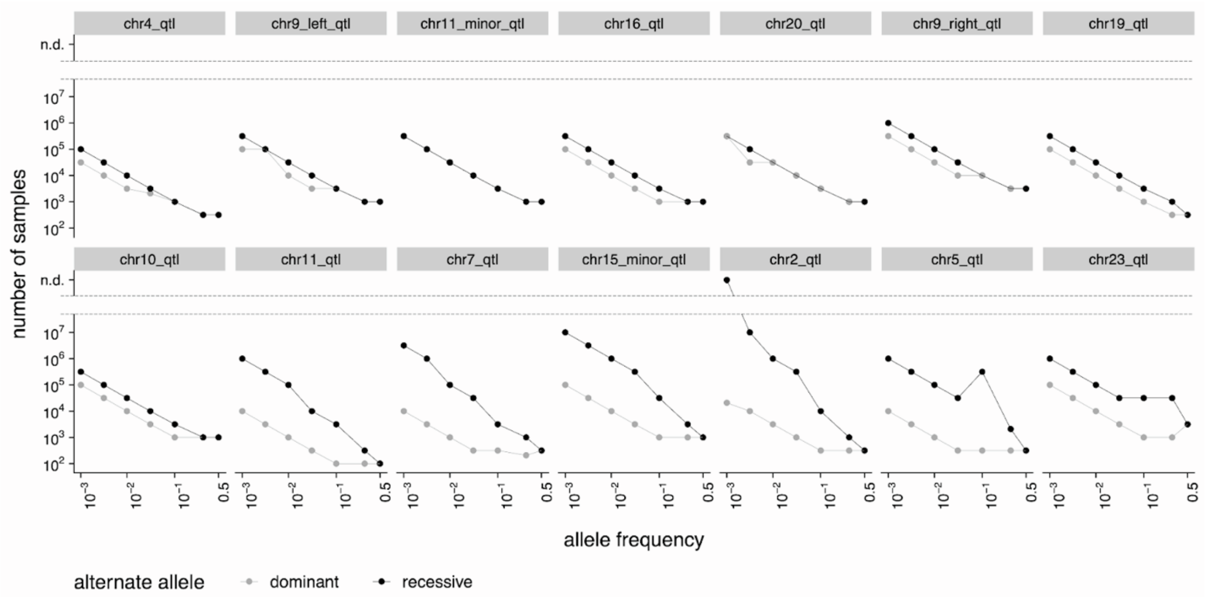

Figure S6. Simulation of the effect of dominance directionality, related to Figure 6. Simulation results analogous to the ones in **Figure 6,** but faceted by simulated QTL. These results use a G + E discovery model, with access to the causal variant, and are generated under a model that does not account for GxG effects. The QTLs are ordered from least to most dominant. In black the estimated sample size requirement for when the alternate allele at the locus is recessive, and in grey the estimated sample size requirement for when the alternate allele is dominant. Notice how the sample size required for discovery is systematically higher when the alternate allele is recessive (the most likely scenario in real populations where high effect recessive alleles are selected against).

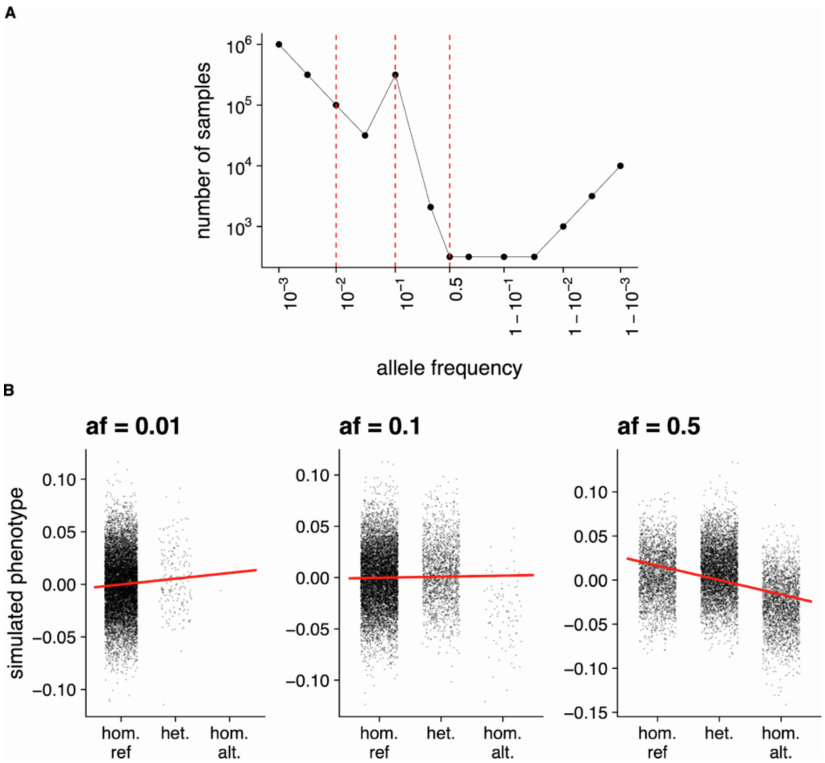

Figure S7. Consequences of overdominance, related to Figure 6. A. Extract from the simulation described in **Figure 6** showcasing an overdominant QTL (chr5_qtl). The results shown use a G + E discovery model, with access to the causal variant, and are generated under a model that does not account for GxG effects. Allele frequencies of 0.01, 0.1, and 0.5 are highlighted with red dashed vertical lines. Notice how at an allele frequency of 0.1 the required sample size increases sharply. **B.** Detailed plot of the sample-level simulation results for the allele frequencies (af) highlighted in panel A. Notice how the effect size estimate (red slope) depends on allele frequency. Going from an allele frequency of 0.01 to 0.5 the slope reverses direction. At an allele frequency of 0.1 this locus is not discoverable under a linear model because the effect of the heterozygous and homozygous samples cancels out almost exactly, leading to the flat slope in panel B and the spike in sample size in panel A.

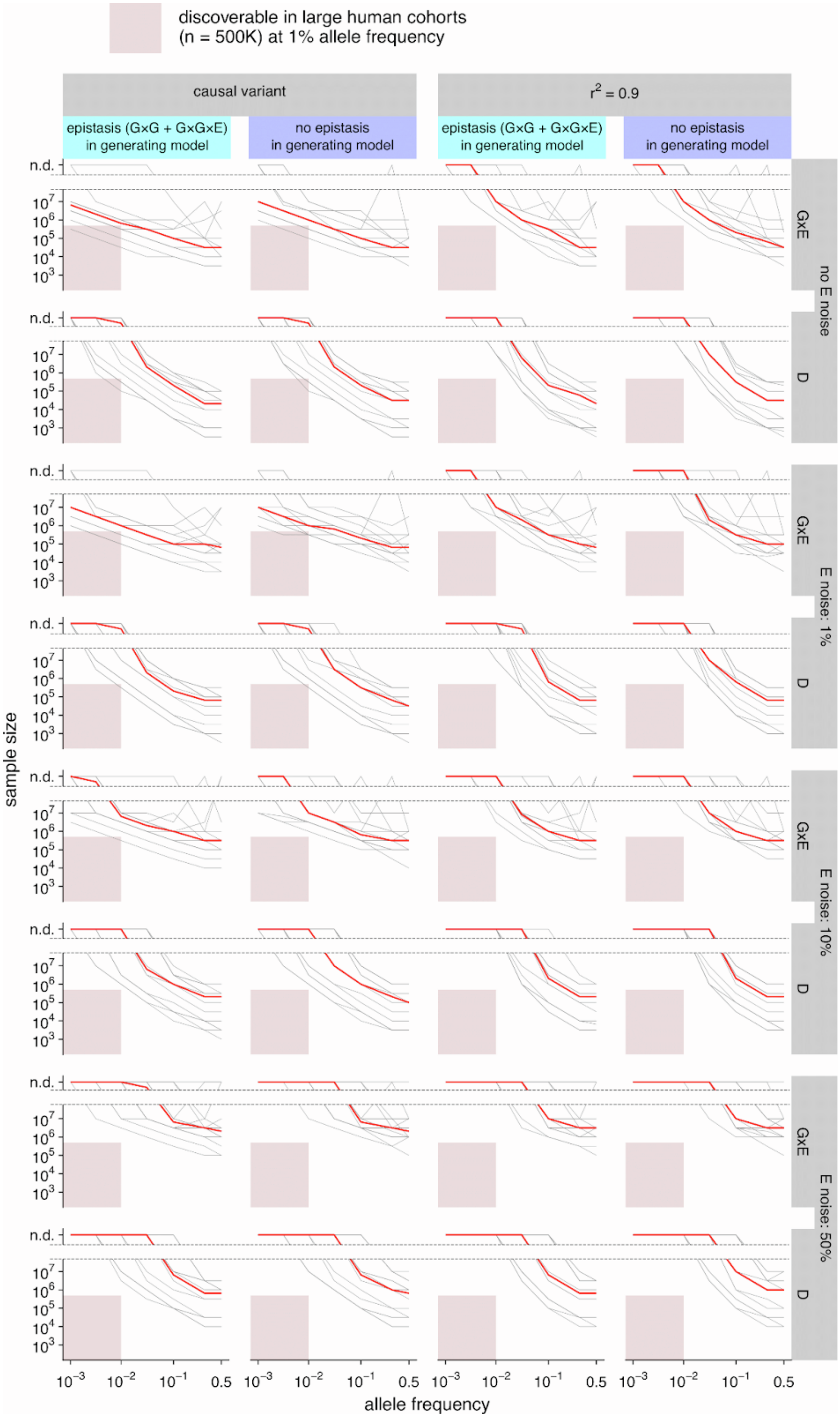

Figure S8. Simulation of the discoverability of non-additive effects, related to Figure 6. Simulation results analogous to the ones in **Figure 6**, but where the discoverability is evaluated against an additive G + E null model and not an E only model. Thus, the minimum sample size estimates reported here refer to what would be required to discover a significant non-additive effect (D: dominance, and GxE: gene-by-environment) when the additive effect is already accounted for.

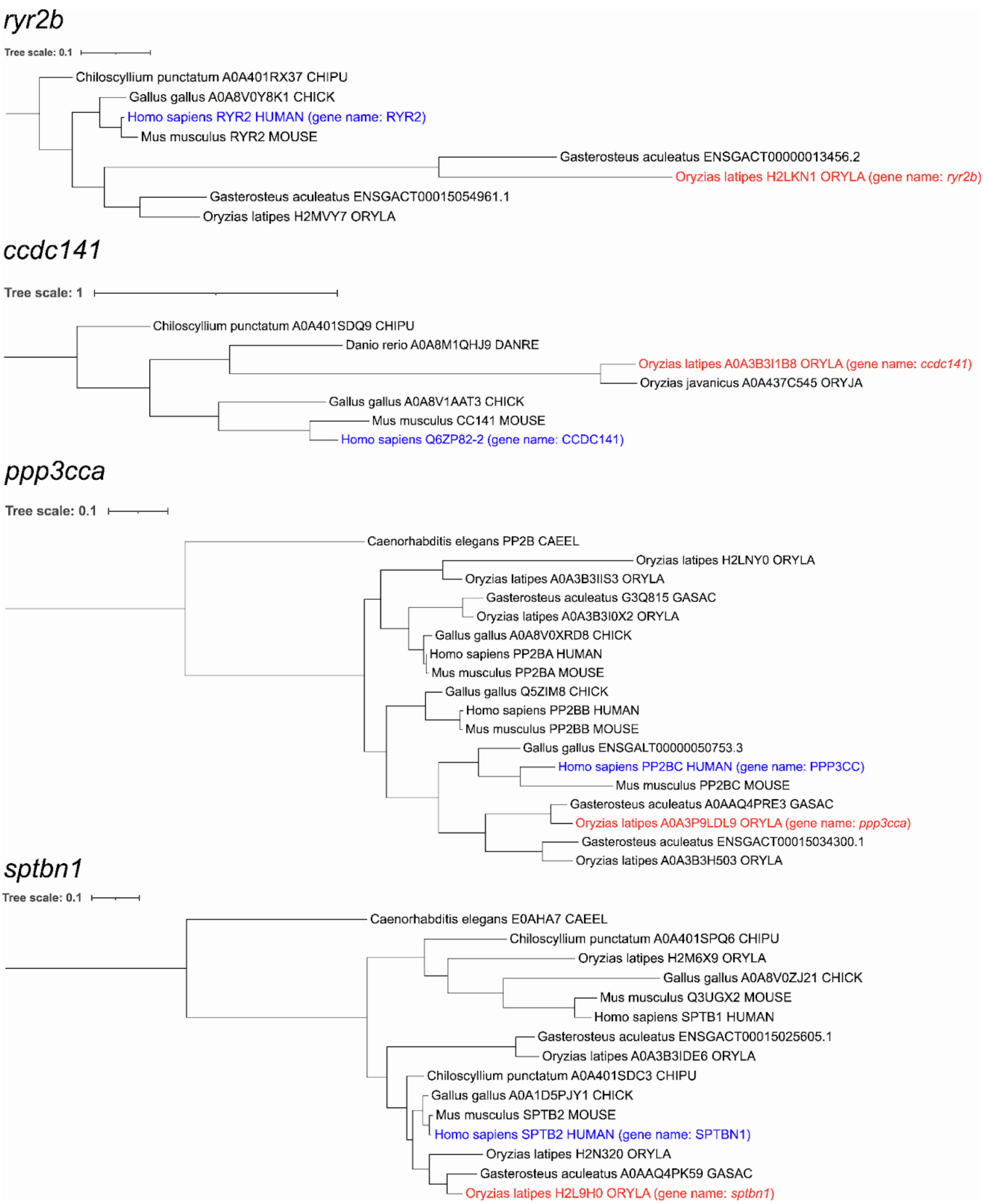

Figure S9. Gene trees for experimentally confirmed candidate genes, related to Figure 5. Gene trees for candidate medaka genes identified via QTL mapping and confirmed by gene editing (in red). Some of the confirmed candidate genes have a complex evolutionary history with multiple paralogues in different species, but in each case it was possible to identify a likely human orthologue or co-orthologue (in blue). Note that the tree identifiers refer to the Uniprot or ENSEMBL IDs used to obtain the protein sequence. The gene names corresponding to the confirmed medaka genes and the corresponding human orthologs or co-orthologs are written in parentheses.

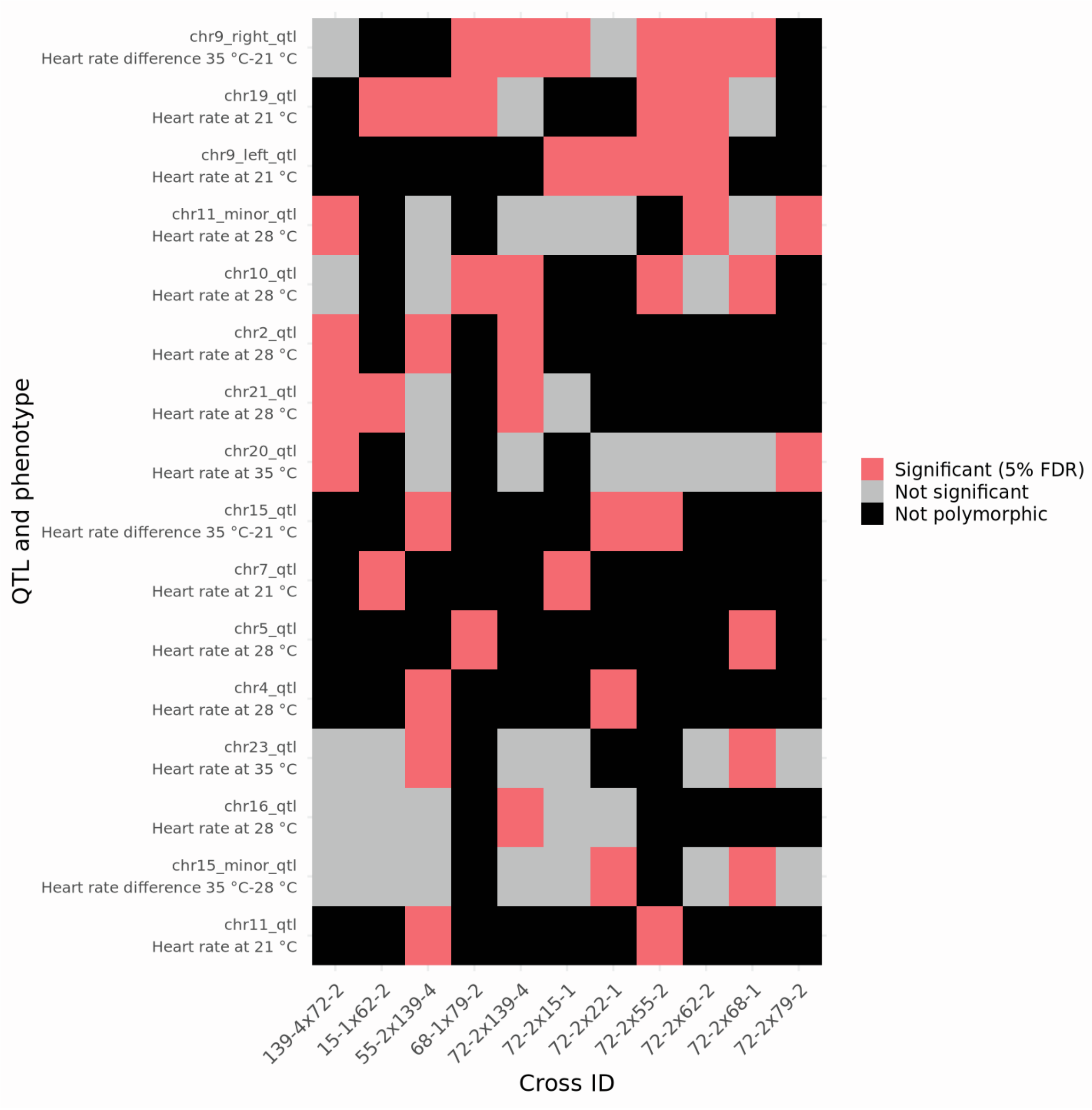

Figure S10. QTL by F2 cross discovery matrix, related to Figure 3. Post-hoc assessment of the 16 QTLs described in this study within individual crosses. Significant associations are reported at a False Discovery Rate (FDR) below 5% across the full matrix. For each QTL, we tested only the phenotype with the strongest signal in the discovery GWAS (lowest p-value), using linear models with additive and dominance terms and the same covariate structure as in the full-scale GWAS. All but one QTL were detected in more than one cross, supporting their validity as genuine association signals. Failure to detect a QTL in a single cross may reflect limited statistical power due to smaller sample size, lack of polymorphism for the causal allele within the cross, differences in linkage disequilibrium between the causal variant and the lead SNP, or epistatic effects. *p*-values: likelihood-ratio test.
